# Effects of Proanthocyanidins on Arsenic Methylation Metabolism and Efflux in Human Hepatocytes L-02

**DOI:** 10.1155/2019/3924581

**Published:** 2019-07-04

**Authors:** Qing-Xin Ren, Meng-Chuan Xu, Qiang Niu, Yun-Hua Hu, Hai-Xia Wang, Shu-Gang Li

**Affiliations:** Department of Public Health, College of Medicine, Shihezi University, Shihezi 832000, Xinjiang, China

## Abstract

This study investigated the effects of proanthocyanidins (PC) on arsenic methylation metabolism and efflux in human hepatocytes (L-02), as well as the relationships between PC and GSH, MRP1 and other molecules. Cells were randomly divided into blank control group, arsenic trioxide exposure group (ATO, As_2_O_3_, 25*μ*mol/L), and PC-treated arsenic exposure group (10, 25, 50mg/L). After 24/48h, the contents of different forms of arsenic were determined, and the methylation indexes were calculated. Intracellular S-adenosyl methionine (SAM), arsenic (+3 oxidation state) methyltransferase (AS3MT), multidrug resistance-associated protein 1 (MRP1), and reduced glutathione (GSH) were ascertained. Changing trends were observed and the correlation between arsenic metabolism and efflux related factors and arsenic metabolites was analyzed. We observed that cells showed increased levels of content/constituent ratio of methyl arsenic, primary/secondary methylation index, methylation growth efficiency/rate, and the difference of methyl arsenic content in cells and culture medium (P<0.05, resp.). Compared with ATO exposure group, the intracellular SAM content in PC-treated group decreased, and the contents of GSH, AS3MT, and MRP1 increased (P<0.05, resp.). There was a positive correlation between the content of intracellular GSH/AS3MT and methyl arsenic. The content of MRP1 was positively correlated with the difference of methyl arsenic content in cell and culture medium; conversely, the SAM content was negatively correlated with intracellular methyl arsenic content (P<0.05, resp.). Taken together, these results prove that PC can promote arsenic methylation metabolism and efflux in L-02 cells, which may be related to the upregulation of GSH, MRP1, and AS3MT levels by PC.

## 1. Introduction

Arsenic (As) is a ubiquitous harmful element in the environment. At present, there are an estimated 2,102 villages in 14 provinces of China ranked higher in arsenic content, with a population of 1.15 million at risk. High arsenic drinking water has also been reported in the United States, Chile, and other countries in the world [1–3]. Arsenic has the effects of bioaccumulation, toxicity, and carcinogenesis and can cause liver [4] and cardiovascular diseases [5] and nervous system damage [6]. When inorganic arsenic enters the organism, it is mainly converted to organic form by methylation in the liver and then excreted out of the organism. This process is catalyzed by arsenic (+3 oxidation state) methyltransferase (AS3MT). Reduced glutathione (GSH) plays a vital role in arsenic methylation metabolism. IAs^5+^ can be reduced to iAs^3+^ by GSH, combining methyl from its donor S-adenosyl methionine (SAM) [7, 8]. After oxidative methylation, iAs is converted into dimethyl arsenate (DMAV) which is less toxic and is excreted in vitro [9]. Furthermore, GSH can interact with multidrug resistance-associated protein 1 (MRP1) and participate in arsenic efflux [10]. MRP1 could promote the cotransportation of As(OH)_3_ with GSH out of cells to reduce intracellular drug concentrations, which may induce drug resistance and limit the efficiency of arsenic derived anticancer or antileukemic activity [11]. Leslie E M et al. [12] proposed MRP1 mediated arsenic efflux through a cotransport mechanism with GSH. Consequently, the exploration of the biological effects of GSH, MRP1, and other factors is of great significance in the prevention and treatment of arsenic poisoning.

Human hepatocytes L-02 originate from human body and are closer to the normal human environment. They are convenient to study the mechanism of drug action. In addition, L-02 cells are easy to survive and have a wide range of adaptation conditions, which can reflect the metabolism of arsenic in the liver. Proanthocyanidins (PC) are natural polyphenolic compounds widely distributed in grape seeds, pine bark, and other plant tissues, which can antagonize arsenic-induced liver oxidative damage by upregulating GSH and other protective proteins [13, 14]. However, it has not been elucidated whether PC can affect arsenic methylation metabolism and efflux by affecting GSH and MRP1. In this study, different doses of PC were designed to treat the cell lines with As_2_O_3_. After a certain period of time, a determination of the contents of related indexes along with an analysis of the effects of arsenic and PC was made. The aim of this study was to explore the effects of PC on arsenic methylation metabolism and efflux in L-02 cells, which may provide a theoretical basis for the application of PC in the prevention and treatment of arsenic poisoning.

## 2. Materials and Methods

### 2.1. Reagents

As_2_O_3_ was purchased from Beijing Chemical Reagent Corp. (Beijing, China). PC which was purified small molecular dimers with purity greater than 98% was obtained from JF-Natural (Tianjin, China). Human Hepatocytes (L-02) were purchased from OBiO Technology (Shanghai) Corp. Fresh fetal bovine serum was acquired from Sijiqing Bioengineering Material Co., Ltd. (Hangzhou, China). Trypsin was purchased from Difco Company (America). KOH was obtained from Shanghai Chemical Reagent Company. KBH_4_ and (NH_4_)_2_HPO4 were purchased from China National Pharmaceutical Group Corp. Sodium monomethyl/dimethyl arsenate standard and As^5+^/As^3+^ ICP-MS standard solution were acquired from American Sigma Company. GSH, ELISA assay kits, phosphate buffer solution (PBS), DMEM cell-culture mediums, syringes, micropipettes, and 96 well enzyme-labeled plates were purchased from Nanjing Jiancheng Bioengineering Institute (Nanjing, China).

### 2.2. Apparatus

A microplate reader (680) was procured from American Bio-Rad Company. A constant temperature water bath (SHA-B) was purchased from Changzhou Guohua Electric Appliance Co., Ltd. (Changzhou, China). A high-speed refrigerated centrifuge (TGL-16G-A) was acquired from Shanghai Anting Scientific Instrument Factory (Shanghai, China). A manual glass homogenizer was purchased from Shanghai Bioengineering Company (Shanghai, China). An inverted phase-contrast microscope (AE31) was obtained from Motic Group Co., Ltd. (Xiamen, China). A pressure steam sterilizer (TX400Z) was bought from Shanghai SANSHEN Medical Instrument Co., Ltd. (Shanghai, China). A super-clean worktable (SW-CJ-2FD) was ordered from Suzhou Purification Equipment Co., Ltd. (Suzhou, China). A high-performance liquid chromatography-hydride generation atomic fluorescence spectrometry analyzer (SA20) was purchased from Beijing Jitian Instrument Co., Ltd. (Beijing, China). A precision electronic balance was acquired from Shanghai Precision and Scientific Instrument Co., Ltd. (Shanghai, China). A CO_2_ incubator (HF151) was purchased from Shanghai LISHEN Scientific Instrument Co., Ltd. (Shanghai, China). A vortex mixer was procured from Shanghai Ya-rong Biochemistry Instrument Factory (Shanghai, China).

### 2.3. Cell Culture

L-02 cells were cultured in DMEM medium containing 10% fetal bovine serum, 0.0625g/L penicillin, and 0.1g/L streptomycin. The culture medium was put into a CO_2_ incubator containing 5% saturated humidity. The temperature was set at 37°C. When cells grew to about 85% ~ 90%, they were processed for digestion with 0.25% trypsin. According to the growth condition, the cells were passaged every 3 to 4 days. Cells in logarithmic growth phase were randomly divided into 5 groups. Through the preliminary experiment, we found that when the intervention dose of ATO was more than 25.0*μ*mol/L, the survival rate of L-02 cells was significantly lower than that in the low gradient dose group in 24/48 hours. So the intervention of 25.0*μ*mol/L ATO can make the survival rate of L-02 cells maintain at a higher level. In this way, we can ensure that our experiments can be carried out and observed obvious results. The grouping is shown in Table 1.

The experimental indexes were detected after 24/48h culture of each group cells. Trypsin (0.5mL) was added to the six-well plates respectively aiming a digestion for 1~2min. When the cells were round and exfoliated, as seen under a microscope, the 2mL complete culture solution was added to each well to terminate digestion. The cells in the six-well plates were collected into the centrifuge tube and separated from the culture solution at 1000 r/min for 5min. Then the cells were washed with PBS 3 times and transferred to the centrifuge tubes (each tube contained about 2.5×10^6^ cells). After resuspension, cells were frozen and thawed repeatedly and centrifuged at 1500 r/min for 15 minutes. Afterward, cells were rinsed with 350*μ*L PBS 3 times, and the supernatant was obtained. The cell-culture solution was collected and filtered with 0.2*μ*m pore membrane. Subsequently, the 0.5mL solution was taken for the measurement.

### 2.4. Determination and Calculation of Arsenic and Its Methylation Metabolites

High-performance liquid chromatography-hydride generation atomic fluorescence spectrometry (HPLC-HGAFS) method was developed for the determination of intracellular and extracellular arsenic with its metabolites. The levels of iAs^3+^, iAs^5+^, monomethylated arsenic (MMA), and dimethylated arsenic (DMA) were detected. Total arsenic (TAs) and the ratios of iAs^3+^, iAs^5+^, MMA, and DMA (iAs^3+^%, iAs^5+^%, MMA%, and DMA %) were calculated. We also calculated primary methylation index (PMI, (MMA+DMA)/TAs×100%), secondary methylation index (SMI, DMA/(MMA+DMA)×100%), monomethylation growth rate ((MMA_48h_+DMA_48h_-MMA_24h_-DMA_24h_)/24), dimethylation growth rate ((DMA_48h_-DMA_24h_)/24), monomethylation growth efficiency ((MMA_48h_+DMA_48h_-MMA_24h_-DMA_24h_)/(TAs_48h_-MMA_24h_-DMA_24h_)×100%), dimethylation growth efficiency ((DMA_48h_-DMA_24h_)/(MMA_48h_+DMA_48h_-DMA_24h_)×100%), and the difference of extracellular and intracellular concentrations of iAs^3+^, iAs^5+^, MMA, and DMA (△iAs^3+^, △iAs^5+^, △MMA, and △DMA).

### 2.5. Methylation Metabolism and Efflux Related Indexes Assay

After 24/48 hours of culture, the cells were tested strictly according to the operation methods of the corresponding apparatus and the instruction manual of the kits. High-performance liquid chromatography (HPLC) was used to determine the content of SAM. AS3MT and MRP1 in cells were determined by ELISA kits, and intracellular GSH was determined by micro ELISA.

### 2.6. Statistical Analysis

The data were extracted by Excel 2010 software and analyzed using SPSS software for Windows version 21.0. The experimental results were expressed as the mean ± standard deviation. Analysis of variance (ANOVA) was used to detect the differences among the experimental groups. Bonferroni method was used in the pairwise comparison, and Pearson correlation analysis was used in relevance analysis. All tests used a significance level of *α*=0.05, and a result of P<0.05 was considered to be statistically significant.

## 3. Results

### 3.1. PC Increased the Contents of Arsenic Metabolites in L-02 Cells Exposed by ATO

**Figure 1:**
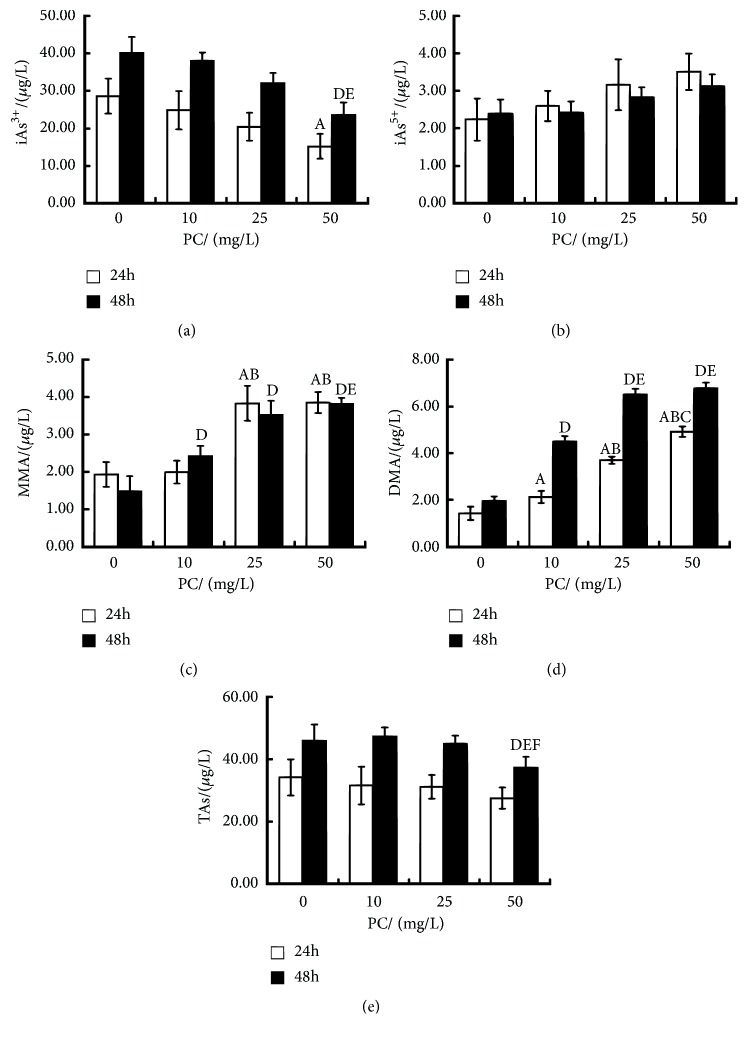
Effects of proanthocyanidins (PC) on inorganic arsenic, arsenic metabolites, and total arsenic (TAs) contents in L-02 cells exposed by arsenic trioxide (ATO). The contents of iAs^3+^ (a), iAs^5+^ (b), MMA (c), DMA (d), and TAs (e) are shown. Values are means (n=3 for each group), with standard deviations represented by vertical bars. ^A^ Indicating significant difference from ATO exposure group with 24h at P<0.05. ^B^ Indicating significant difference from PC (10mg/L)-treated group with 24h at P<0.05. ^C^ Indicating significant difference from PC (25mg/L)-treated group with 24h at P<0.05. ^D^ Indicating significant difference from ATO exposure group with 48h at P<0.05. ^E^ Indicating significant difference from PC (10mg/L)-treated group with 48h at P<0.05. ^F^ Indicating significant difference from PC (25mg/L)-treated group with 48h at P<0.05. iAs, inorganic arsenic; MMA, monomethylated arsenic; DMA, dimethylated arsenic.

The effects of ATO and PC on the contents of arsenic and its metabolites are shown in Figure 1. After 24 hours of intervention, the contents of MMA and DMA were significantly higher in PC (10, 25, 50mg/L)-treated group than in the ATO exposure group. In comparison with PC (10, 25mg/L)-treated group, the treatment of PC (50mg/L) caused an increase of DMA. However, PC (50mg/L)-treated group decreased the level of iAs^3+^ (P<0.05, resp.). After 48 hours of intervention, the change trend of each index was basically the same as that of 24h intervention. The TAs content in PC (50mg/L)-treated group was less than that in ATO exposure group and PC (10, 25mg/L)-treated group (P<0.05, resp.).

### 3.2. PC Increased the Ratio of Arsenic Metabolites in L-02 Cells Exposed by ATO

**Figure 2:**
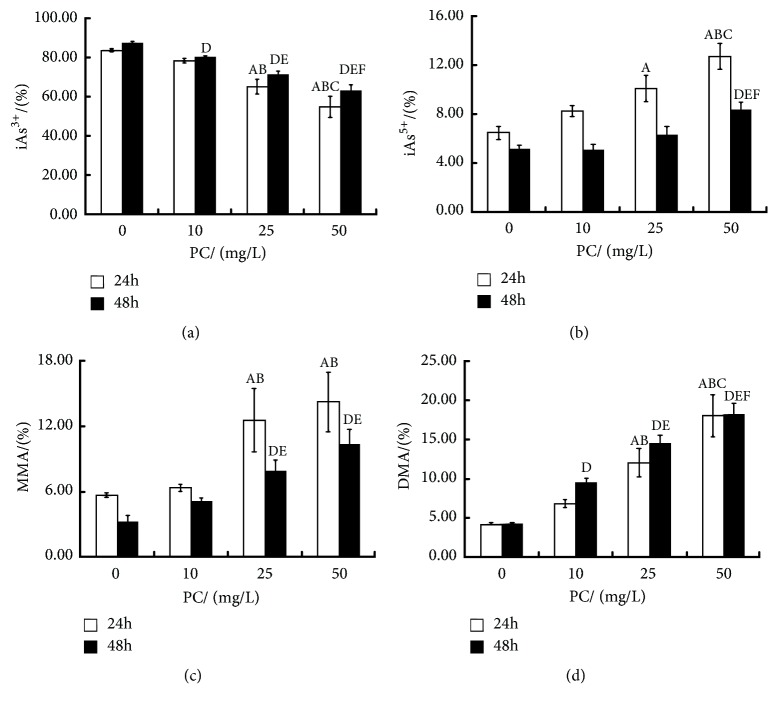
Effects of proanthocyanidins (PC) on constituent ratios (%) of various forms of arsenic in L-02 cells exposed by arsenic trioxide (ATO). The constituent ratios of iAs^3+^ (a), iAs^5+^ (b), MMA (c), and DMA (d) are shown. Values are means (n=3 for each group), with standard deviations represented by vertical bars. ^A^ Indicating significant difference from ATO exposure group with 24h at P<0.05. ^B^ Indicating significant difference from PC (10mg/L)-treated group with 24h at P<0.05. ^C^ Indicating significant difference from PC (25mg/L)-treated group with 24h at P<0.05. ^D^ Indicating significant difference from ATO exposure group with 48h at P<0.05. ^E^ Indicating significant difference from PC (10mg/L)-treated group with 48h at P<0.05. ^F^ Indicating significant difference from PC (25mg/L)-treated group with 48h at P<0.05. iAs, inorganic arsenic; MMA, monomethylated arsenic; DMA, dimethylated arsenic.

After 24 hours of intervention, compared with ATO exposure group, the constituent ratio of iAs^3+^ decreased and the constituent ratios of iAs^5+^, MMA, and DMA increased with the increasing PC dosage (P<0.05, resp.). The trend of 48h-intervention was basically the same as that of 24h. See Figure 2.

### 3.3. PC Improved the Level and Capacity of Methylation in L-02 Cells Exposed by ATO

**Figure 3:**
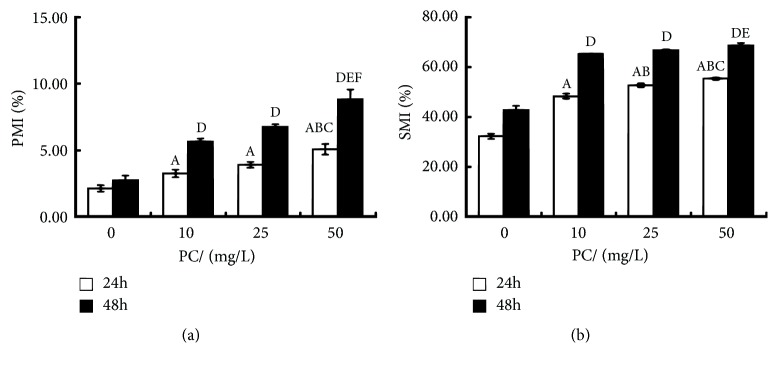
Effect of proanthocyanidins (PC) on primary methylation index (PMI) and secondary methylation index (SMI) in L-02 cells exposed by arsenic trioxide (ATO). The PMI (a) and SMI (b) are shown. Values are means (n=3 for each group), with standard deviations represented by vertical bars. ^A^ Indicating significant difference from ATO exposure group with 24h at P<0.05. ^B^ Indicating significant difference from PC (10mg/L)-treated group with 24h at P<0.05. ^C^ Indicating significant difference from PC (25mg/L)-treated group with 24h at P<0.05. ^D^ Indicating significant difference from ATO exposure group with 48h at P<0.05. ^E^ Indicating significant difference from PC (10mg/L)-treated group with 48h at P<0.05. ^F^ Indicating significant difference from PC (25mg/L)-treated group with 48h at P<0.05.

**Figure 4:**
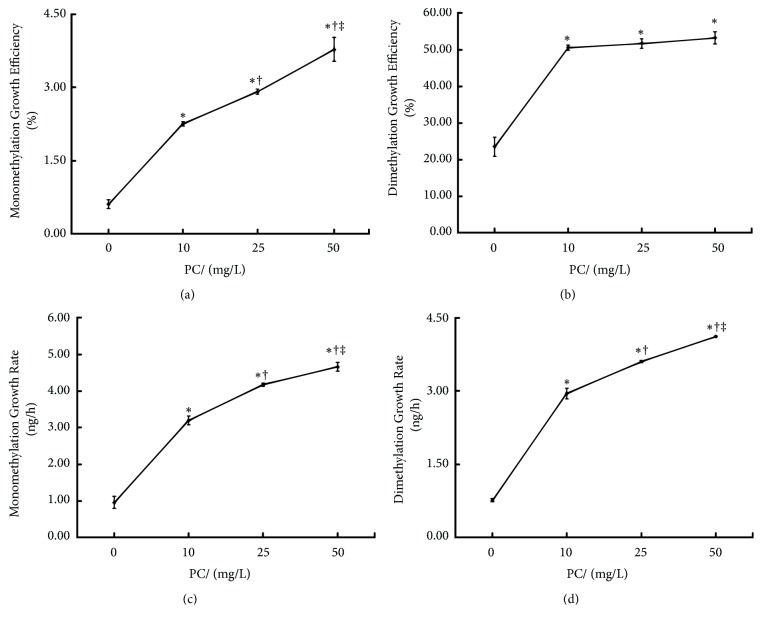
Effects of proanthocyanidins (PC) on methylation growth rate (ng/h) and efficiency (%) of arsenic in L-02 cells exposed by arsenic trioxide (ATO) within 24~48 hours. The monomethylation growth efficiency (a), dimethylation growth efficiency (b), monomethylation growth rate (c), and dimethylation growth rate (d) are shown. Values are means (n=3 for each group), with standard deviations represented by vertical bars. ^*∗*^Indicating significant difference from ATO exposure group at P<0.05; ^†^indicating significant difference from PC (10mg/L)-treated group at P<0.05; ^‡^indicating significant difference from PC (25mg/L)-treated group at P<0.05.

After 24/48h, the PMI and SMI levels in all PC-treated groups increased compared with the ATO exposure group (P<0.05, resp.). The SMI of PC (25mg/L)-treated group was higher than that of PC (10mg/L)-treated group, and the PMI and SMI of PC (50mg/L)-treated group were higher than those of PC (10, 25mg/L)-treated group (P<0.05, resp.). See Figure 3. As shown in Figure 4, within 24~48 hours of intervention, the methylation growth rate and efficiency in each PC-treated group were higher than those in the ATO exposure group (P < 0.05, resp.), showing an upward trend with the increasing PC dosage.

### 3.4. PC Upregulated the Levels of GSH and AS3MT in L-02 Cells Exposed by ATO

**Figure 5:**
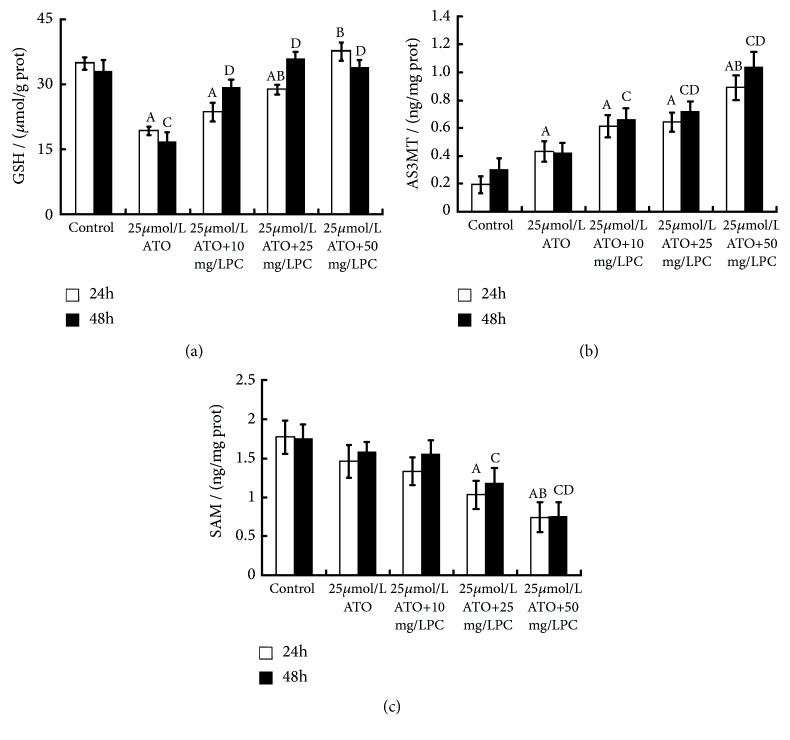
Effects of proanthocyanidins (PC) on the levels of glutathione (GSH, *μ*mol/g), arsenic (+3 oxidation state) methyltransferase (AS3MT, ng/mg) and S-adenosyl methionine (SAM, ng/mg) in L-02 cells exposed by arsenic trioxide (ATO). The GSH (a), AS3MT (b), and SAM (c) are shown. Values are means (n=3 for each group), with standard deviations represented by vertical bars. ^A^ Indicating significant difference from the blank control group with 24h at P<0.05. ^B^ Indicating significant difference from ATO exposure group with 24h at P<0.05. ^C^ Indicating significant difference from the blank control group with 48h at P<0.05. ^D^ Indicating significant difference from ATO exposure group with 48h at P<0.05.

We found out that the intracellular content of GSH in ATO exposure group was lower than that in the blank control group and the AS3MT content was higher than that in the blank control group after 24/48h of intervention (P<0.05, resp.). The contents of GSH and AS3MT in the PC-treated group were higher than those of ATO exposure group, and the content of SAM was lower than that of ATO exposure group (P<0.05, resp.). See Figure 5.

### 3.5. The Contents of GSH and AS3MT Were Positively Correlated with Arsenic Metabolites and the Contents of SAM Were Negatively Relevant to Them

**Figure 6:**
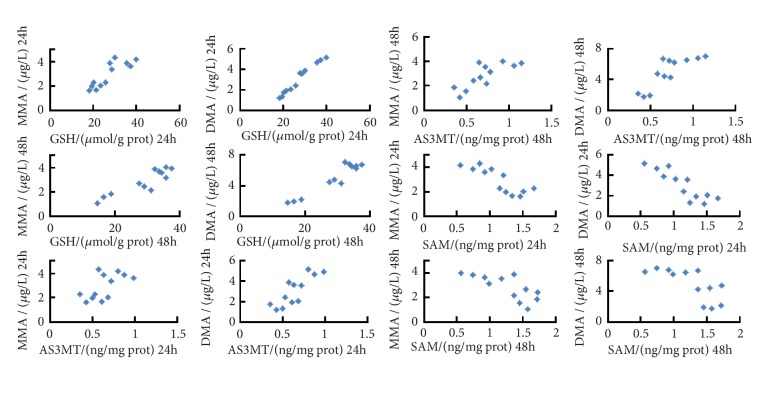
Analysis of the correlation between glutathione (GSH)/arsenic (+3 oxidation state) methyltransferase (AS3MT)/S-adenosyl methionine (SAM) and arsenic metabolites contents in arsenic trioxide (ATO) infected L-02 cells by proanthocyanidins (PC) intervention. The horizontal and vertical coordinates of each data point referred to the contents of GSH, AS3MT, SAM, and corresponding methyl arsenic under the intervention of different doses of PC. MMA, monomethylated arsenic; DMA, dimethylated arsenic.

We made the correlation between the protein contents of GSH, AS3MT, and SAM and different forms of arsenic content in L-02 cells. As shown in Figure 6, we discovered that MMA and DMA contents were positively correlated with GSH and AS3MT contents and negatively correlated with SAM content (P < 0.05, resp.).

### 3.6. PC Upregulated the Levels of △MMA and △DMA and Downregulated the Levels of △iAs^*3*+^ and △iAs^*5*+^

After 24/48 hours of intervention, the △MMA of PC (50mg/L)-treated group was higher than that of ATO exposure group (P<0.05), and the △DMA of each PC-treated group was higher than that in ATO exposure group (P<0.001, resp.), as shown in Table 2. In comparison with the ATO exposure group, the treatment of PC (50mg/L) caused a decrease in △iAs^3+^ (P=0.002). In addition, the △iAs^5+^ in PC (50mg/L)-treated group was lower than that in ATO exposure group after the period of 24h (P=0.009). See Table 3.

### 3.7. PC Upgraded the Level of MRP1 in L-02 Cells Exposed by ATO

**Figure 7:**
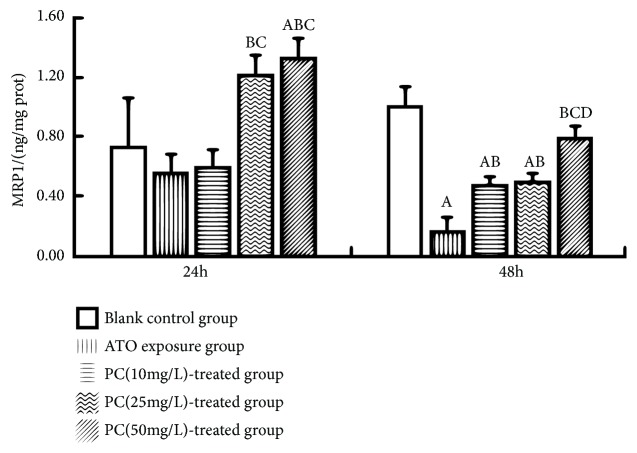
Effects of proanthocyanidins (PC) on the contents of multidrug resistance-associated protein 1 (MRP1, ng/mg) in L-02 cells exposed by ATO. Values are means (n=3 for each group), with standard deviations represented by vertical bars. ^A^ Indicating significant difference from the blank control group at P<0.05. ^B^ Indicating significant difference from ATO exposure group at P<0.05. ^C^ Indicating significant difference from PC(10mg/L)-treated group at P<0.05. ^D^ Indicating significant difference from PC(25mg/L)-treated group at P<0.05.

Compared with the blank control group, MRP1 decreased in 48h-ATO exposure group (P<0.001). The level of intracellular MRP1 in the PC (25, 50 mg/L)-treated group for 24 hours was higher than that in the ATO exposure group (P<0.05, resp.). After 48 hours of intervention, the MRP1 level of each PC-treated group was higher than that of ATO exposure group (P<0.05, resp.) and increased with the increasing PC dosage. See Figure 7.

### 3.8. The Content of MRP1 Was Positively Relevant to the Contents of △MMA and △DMA

**Figure 8:**
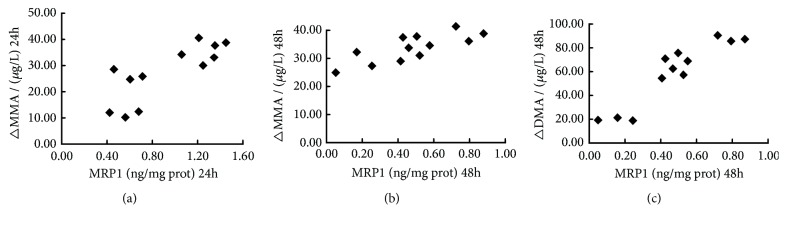
Analysis of the correlation between multidrug resistance-associated protein 1 (MRP1) contents and the difference of extracellular and intracellular concentrations of monomethylated arsenic (△MMA)/dimethylated arsenic (△DMA).

There was a positive correlation between MRP1 content and △MMA after 24h-intervention (P=0.002), and the content of MRP1 was positively correlated with △MMA and △DMA after 48h-intervention (P<0.05, resp.), as shown in Figure 8.

## 4. Discussion

Arsenic is a hazardous element that seriously endangers public health. It widely exists in nature and can cause damage to tissues and organs [15, 16]. The methylation metabolism of arsenic is one of the most crucial ways of its toxicity, which is related to GSH, AS3MT, and SAM. GSH can interact with MRP1 to participate in the efflux of arsenic [17]. PC is a kind of natural polyphenolic compounds with strong antioxidant activity widely found in grape seeds and other plant tissues. It can upregulate the levels of methylation-related molecules such as GSH. The results showed that PC could increase the indexes and rates of arsenic methylation and the contents of AS3MT and MRP1 in ATO-exposed L-02 cells, suggesting that PC might promote the methylation metabolism and efflux of arsenic in L-02 cells.

Studies have shown that PC can antagonize arsenic-induced oxidative damage in hepatocytes [18]. PMI and SMI are classical indicators to measure the level of arsenic methylation, reflecting the ability of the first methylation of inorganic arsenic to produce methyl arsenic, and the ability of the second methylation to convert MMA into DMA, respectively [19]. In order to avoid the effects of MMA and DMA accumulated in 24 hours on the calculated values of PMI_48h_ and SMI_48h_ and their relationships with PC dosage, the monomethylation and dimethylation growth efficiency measured in this study represent the intracellular conversion levels of inorganic arsenic to methyl arsenic and MMA to DMA in 24~48 hours. It was found out that PMI and SMI and the efficiency and rate of methylation growth increased with the increase of PC dosage. In addition, with the PC treatment, the content and constituent ratio of inorganic arsenic decreased, while those of methyl arsenic increased, which indicated that PC promoted the methylation of arsenic in L-02 cells. GSH and AS3MT are helpful to the metabolism of arsenic methylation, and SAM can provide methyl for inorganic arsenic [20, 21]. Compared with the blank control group, the content of GSH decreased and the content of AS3MT increased in ATO-exposed group, which was consistent with the results of Hu Yu [22] and Wu Jun [23]. We hypothesize that arsenic could consume intracellular GSH and induce the defensive response of AS3MT. The increased levels of GSH and AS3MT under PC intervention may be related to the activation of phosphoinositide 3-kinase (PI3K)/protein kinase B (PKB/Akt) signal pathway with antioxidant effect [24]. It has also been reported that GSH modulates AS3MT activity [25]. Hence, we speculate that the upregulation of GSH promotes the transfer of methyl from SAM to arsenic in various forms catalyzed by AS3MT to complete methylation metabolism.

MRP1 is a member of adenosine triphosphate binding cassette (ABC) transporter superfamily, which can transport intracellular substance extracellularly in reverse concentration gradients, and is widely distributed in the organism as a kind of GSH transport pump [26, 27]. The results showed that the intracellular TAs decreased with the increasing PC dosage, and the △MMA and △DMA in the PC intervention group were higher than those in the ATO exposure group. The content of MRP1 in ATO-exposed cells was lower than that in the blank control group, but increased after PC intervention, showing a dose-response relationship with PC. The content of MRP1 was positively correlated with △MMA and △DMA. This suggests that MRP1 contributes to the efflux of arsenic from cells, and PC promotes the expression of MRP1. Among several forms of arsenic, DMA has less toxicity and discharges from the organism easily [9, 28]. Therefore, PC can promote arsenic methylation metabolism, which in turn promotes arsenic efflux. Alternatively, PC can antagonize arsenic-induced apoptosis [18, 29]; therefore, PC can affect more active cells to participate in arsenic metabolism and contribute to arsenic efflux. Previous studies have shown that nuclear factor E2 related factor 2 (Nrf2)-antioxidant response element (ARE) signaling pathway could antagonize arsenic-induced oxidative damage [30] and can also upregulate the levels of GSH [31] and MRP1 [32]. Thus, we presume that PC activates this pathway and promotes the expression of MRP1 and GSH. At the same time, GSH upregulates the level of AS3MT and promotes arsenic methylation metabolism. And we speculate that arsenic and its metabolites form complexes with GSH. MRP1 binds to the arsenic-GSH complexes and consumes ATP to pump arsenic out of the cells [33]. It can be seen that PC antagonizes the toxicity of arsenic by promoting the methylation metabolism and efflux of it. Whether PC can prevent arsenic poisoning remains to be further studied.

## 5. Conclusions

In conclusion, PC can promote arsenic methylation metabolism and efflux in L-02 cells, which may be related to the upregulation of GSH, MRP1, and AS3MT levels by PC. However, this study is a cell-based experiment, and further investigation in different cell lines and in vivo is needed to clarify these findings. This way our findings could help provide a better understanding of the mechanism and achieve better development and utilization of PC. Moreover, we can also provide a theoretical basis for preventing arsenic poisoning and improving public health.

## Figures and Tables

**Table 1:** Treatment measures and grouping.

Group	Treatment measures
Blank control group	As_2_O_3_ + PC (0*μ*mol/L + 0mg/L)
ATO exposure group	As_2_O_3_ + PC (25*μ*mol/L + 0mg/L)
PC-treated group	As_2_O_3_ + PC (25*μ*mol/L + 10mg/L)
PC-treated group	As_2_O_3_ + PC (25*μ*mol/L + 25mg/L)
PC-treated group	As_2_O_3_ + PC (25*μ*mol/L + 50mg/L)

Note: PC, proanthocyanidins; ATO, arsenic trioxide.

**Table 2:** Effects of proanthocyanidins (PC) on the changes of the difference of extracellular and intracellular concentrations of methyl arsenic (n=3).

Group	△MMA (*μ*g/L)		△DMA (*μ*g/L)	
	24h	48h	24h	48h
ATO (25*μ*mol/L)	25.62±2.08	28.46±3.76	11.63±1.47	20.18±1.40
ATO + PC (25*μ*mol/L + 10mg/L)	29.35±1.52	31.87±2.36	27.13±2.01^*∗*^	59.74±3.40^*∗*^
ATO + PC (25*μ*mol/L + 25mg/L)	29.11±1.53	36.34±2.02	33.12±2.41^*∗*#^	73.07±2.48^*∗*#^
ATO + PC (25*μ*mol/L + 50mg/L)	32.51±1.72^*∗*^	39.06±2.87^*∗*^	40.01±1.85^*∗*#△^	87.23±2.47^*∗*#△^

Note: the results were described as mean ± SD. ^*∗*^Indicating significant difference from ATO exposure group at P<0.05; ^#^indicating significant difference from PC (10mg/L)-treated group at P<0.05; ^△^indicating significant difference from PC (25mg/L)-treated group at P<0.05. △MMA, the difference of extracellular and intracellular concentrations of monomethylated arsenic; △DMA, the difference of extracellular and intracellular concentrations of dimethylated arsenic; ATO, arsenic trioxide.

**Table 3:** Effects of proanthocyanidins (PC) on the changes of the difference of extracellular and intracellular concentrations of inorganic arsenic (n=3).

Group	△iAs^3+^ (*μ*g/L)		△iAs^5+^ (*μ*g/L)	
	24h	48h	24h	48h
ATO (25*μ*mol/L)	1496.72±48.06	1457.89±25.77	372.28±16.58	361.85±36.03
ATO + PC (25*μ*mol/L + 10mg/L)	1440.92±31.28	1305.08±42.56	354.78±16.77	315.03±38.53
ATO + PC (25*μ*mol/L + 25mg/L)	1391.13±34.46	1310.49±14.06	335.11±40.96	317.90±26.00
ATO + PC (25*μ*mol/L + 50mg/L)	1286.91±39.73^*∗*#^	1129.89±94.14^*∗*#△^	246.43±44.77^*∗*#^	276.62±23.17

Note: the results were described as mean ± SD. ^*∗*^Indicating significant difference from ATO exposure group at P<0.05; ^#^indicating significant difference from PC (10mg/L)-treated group at P<0.05; ^△^indicating significant difference from PC (25mg/L)-treated group at P<0.05. △iAs, the difference of extracellular and intracellular concentrations of inorganic arsenic; ATO, arsenic trioxide.

## Data Availability

The data used to support the findings of this study are available from the corresponding author upon request.
